# Executive Dysfunction and Language Deficits in a Pediatric Patient with OCD and MDD with Suicidality

**DOI:** 10.1155/2019/6574304

**Published:** 2019-04-30

**Authors:** Melissa Yuan

**Affiliations:** New York Presbyterian Hospital Westchester Division, White Plains, NY, USA

## Abstract

The role of neuropsychiatric testing in psychiatric disorders is becoming more prominent. Neuropsychological measures that are similar across symptom domains and phenomena such as suicidality may help clinicians guide treatment and tailor therapies to the patient in the most effective way possible. We report the case of a 16-year-old girl who presented with bizarre, intrusive suicidal thoughts in the setting of OCD and MDD. This case is unusual in that we have accurate neuropsychological determination of our patient's language and executive function deficits, and we propose a link between them and her expression of suicidality in the context of OCD and MDD.

## 1. Introduction

In recent years, increasing focus is being turned towards defining patterns of neuropsychological dysfunction that are associated with specific mental illness and specific symptom clusters, including bipolar disorder, attention-deficit hyperactivity disorder, posttraumatic stress disorder, and schizophrenia [1–5]. This is largely due to a shift towards investigating brain structure and function underlying psychiatric disorders [6]. An example of this is the attempt to define the neuropsychological attributes of people who attempt suicide. In adults, it has been found that suicidal ideation may result from dysfunctional frontal lobe executive decision‐making [7]. Additionally, previous studies have also found that reduced verbal fluency is more common in suicide attempters than a comparable control group [8].

In addition to defining phenomena such as suicidality, finding neuropsychological measures that are similar across various disorders is of interest. For instance, executive dysfunction is considered to be a state-dependent feature of major depressive disorder (MDD), a state or trait-dependent feature of obsessive-compulsive disorder (OCD), and a possible endophenotype for suicidality. However, the field continues to lack a clear consensus on what these neuropsychiatric “signatures” consist of, especially in the less-studied pediatric psychiatric population, nor has the utility of these been established. For example, there is mixed literature regarding executive function in children with OCD. Beers et al. found that recently diagnosed children with OCD did not perform worse than healthy controls on neuropsychological testing [9]. This finding was replicated by Abramovitch et al., who found that pediatric patients with OCD do not have significant neuropsychiatric performance deficits [10].

It has been postulated that children with disruptive behavior disorders may have poorer receptive, pragmatic, and expressive language skills [11]. This has been shown in attention-deficit hyperactivity disorder and other disruptive behavior disorders, in the pediatric population [12–14]. This has been shown less consistently in OCD and other disorders [15].

We present a pediatric patient with comorbid MDD and OCD with intrusive suicidal thoughts, who had measurable deficits in executive function and language fluency on neuropsychological testing. We believe that this is a timely report of a complex patient that highlights the neuropsychiatric correlates of various psychiatric symptoms and how this can inform treatment.

## 2. Case Presentation

A 16-year-old female with a history of OCD, MDD, and suicidal thoughts, with no past psychiatric hospitalizations, was brought to the psychiatric emergency department for two months of constant suicidal thoughts. She reported that these thoughts occurred “at night when I go to sleep and when I wake up; I am always wanting to die”. However, she felt strongly that these were not her own thoughts and that she did not actually want to commit suicide. On admission, she endorsed dysphoria, anhedonia, initial insomnia, hopelessness, worthlessness, and feelings of guilt. She also expressed “wanting to strangle the ghost out of her throat” in addition to other bizarre, intrusive suicidal and self-harming thoughts.

She endorsed a history of OCD, diagnosed in high school, and severe hypochondriasis since childhood. She has “always” had fears of being ill or contaminated and engaged in cleaning rituals. Her mother reported that she tended to phrase her obsessions in an “odd” way, for example, stating that she was “scared of breathing the air because it is heavy and dirty and full of sickness” or that she wanted to wash “slimy eels” off her hands after she touched a public handrail. This patient also had a history of depressive symptoms starting at age 7, including periodic irritability, anhedonia, difficulty in concentrating, insomnia, and worthlessness. Despite these difficulties, she was doing well in school and succeeded at playing the violin at a high level. The previous two months had been her first time having such severe, persistent suicidal thoughts.

We began treating this patient with 35 mg fluoxetine q.d. for depression and obsessive thoughts, 300 mg gabapentin q.d. for anxiety, and 150 mg quetiapine q.d. for intrusive and bizarre thoughts. In other words, this was a comprehensive treatment regimen with the goal of addressing our patient's OCD and MDD in addition to her suicidality. Her two-month hospital course was complicated by numerous episodes of heightened anxiety and suicidal thoughts, during which she was “terrified” she was going to “need to kill herself to get this ghost out”. She was worried she would act on this thought, reacting with tearfulness, feelings of hopelessness, difficulty in redirecting her emotions, and inability to concentrate on anything else. Some of her depressive symptoms such as dysphoria and insomnia decreased over her stay, but her suicidality and recurrent thoughts continued to be present. Added to the complex nature of this case was the patient's difficulty in expressing the feelings underlying her suicidal thoughts. She continuously said that these thoughts of strangling herself “came from nowhere”, and she could not pinpoint any correlation of these thoughts with her emotional state, level of distress, or feelings of hopelessness. She sometimes stated: “I want to tie something around my neck and I want to die”, or “life is so pointless”, while continuously denying to staff that she had any desire to commit suicide. The patient's family history was notable for OCD in a maternal grandfather. Of interest, the patient's identical twin sister has not had any psychiatric illnesses.

We sent this patient for neuropsychiatric testing upon recommendation from a child specialist. Neuropsychological examination found deficits in executive functioning, as assessed by a Key Search Task and the Virtual Continuous Performance Test, in addition to decreased verbal cognitive performance as assessed by the Verbal Fluency Test, Verbal Fluency Controlled Oral Word Association Test, and the Digit Span and Letter‐Number sequencing subtests of the WAIS‐III. The other portions of the neuropsychological exam included the Test of Variables of Attention, to assess attention; the Wechsler Memory Scale, to assess memory; the Rey-Osterrieth Complex Figure task, to assess visuospatial skills; and the Wechsler Adult Intelligence Scale, to assess general cognitive ability. The results of these other tests were all within normal limits.

The results of this testing led firstly to increased understanding by the patient's family of her mental illness; given that she had been high functioning, her parents had not understood why she was so “crazy”. The relatively objective results of this testing alleviated this pressure on our patient from her family. In addition, further treatment focused more on supporting and encouraging expression of emotion, with more time allotted for the patient to think through responses. The patient and her family were advised to increase levels of external support to help with communication and planning upon discharge.

The patient attended a psychiatric day program before returning home to be followed closely as an outpatient for the past several months. During this time, she has received standard-of-care treatment for MDD and OCD, including fluoxetine q.d. for depression, clomipramine q.d. for OCD, and cognitive behavioral therapy. Concurrently, we have worked in particular to improve her executive function and verbal abilities. The patient completes computerized working-memory training tasks daily and is being coached in mindfulness meditation, two methods that have been shown to increase executive functioning in children [16]. She also has engaged in auditory training exercises to improve verbal processing and memory. Currently, after more than 3 months of this additional training targeted towards neuropsychological deficits in addition to medications and therapy, this patient reports significant improvement. She has more self-efficacy and hope surrounding her mental illness and feels that she “knows what to do” if she feels that way again in the future.

## 3. Discussion

The neurological basis and psychosocial causality of the patient's multiple comorbid disorders are unclear. We propose that executive dysfunction and difficulties with communication led to her unique presentation and bizarre explanations of her intrusive thoughts. It is also possible that her neuropsychological deficits contributed to her protracted depressive episodes and worse outcome relative to her twin sister. It is likely that the combination of her mental rigidity and perseverance, a function of executive dysfunction, and difficulties in communicating verbally, a function of her language abilities, is what led to our patient's unique presentation and difficult treatment course.

Of interest, this patient exhibited impairments in executive functioning and verbal abilities, which is not entirely consistent with neuropsychology testing results in either MDD or OCD [17]. Specifically, MDD is associated with executive function deficits as in our patient, but also impaired psychomotor speed and memory [17]. Similarly, patients with OCD tend to perform worse than healthy controls most consistently on working-memory tasks, but also on attention, executive function, and processing speed neuropsychological tasks in various studies [10, 18]. However, across the literature, there is not yet a consensus as to the clinical significance of these findings. Rather, exploration of neuropsychological functioning in various mental illnesses continues as a crucial part of understanding the entire biopsychosocial picture of a disease.

In particular, neuropsychological testing can provide psychiatrists with a deeper understanding of the cognitive and language impairments cooccurring with psychiatric disorders and may even allow more accurate prognostication of the presentation and course of the illness. Moreover, neuropsychological data may be used to develop treatment strategies tailored specifically for the patient's strengths and weaknesses, as in our patient [6]. It is our hope that this report demonstrates the role that neuropsychiatric testing can play in psychiatric care, and can provide insight into clinical decision-making in children presenting similarly to our patient. Moreover, we believe that more emphasis on understanding neuropsychological underpinnings of behaviors and disorders will help develop our knowledge of the underlying circuitry and perhaps ultimately open new avenues for treatment.
